# Heparin Binding Protein in Sepsis—A Comprehensive Overview of Pathophysiology, Clinical Usage and Utility as Biomarker

**DOI:** 10.3390/biomedicines13092315

**Published:** 2025-09-22

**Authors:** Foteini Tasouli, Eleni Georgopoulou, Christodoulos Chatzigrigoriadis, Dimitrios Velissaris, Christos Michailides

**Affiliations:** Department of Internal Medicine, University Hospital of Patras, 26504 Patras, Greece; tasoulif@gmail.com (F.T.); eleni.geor.11@gmail.com (E.G.); xatzhgrhgoriadhw@gmail.com (C.C.); dvelissaris@upatras.gr (D.V.)

**Keywords:** heparin binding protein, HBP, sepsis, diagnosis, prognosis, biomarkers

## Abstract

The heparin-binding protein (HBP) is an enzymatically inactive protein of the serine protease family that plays an important role in host response to stress, especially infection and sepsis. It is produced by activated neutrophils due to a variety of stimuli and is part of the immune response that leads to macrophage, lymphocyte, and neutrophil activation and monocyte adhesion. Its most common repository is the azurophilic granules of the neutrophils. HBP has been studied as a biomarker for several infections, including central nervous system infection, respiratory tract infection, and urinary tract infection, and in several settings, including the Emergency Department and Intensive Care Unit, with promising results. As a biomarker for infection and sepsis, HBP has been compared to other commonly used biomarkers such as Neutrophil to Lymphocyte Ratio, White Blood Count, C-reactive protein, and Procalcitonin, with at least comparable performance. Its sharp increase is promising for the early detection of sepsis. The ability to differentiate inflammatory conditions from infections and bacterial from non-bacterial causes of infection has also been demonstrated. The sepsis-related organ damage, as it is represented by the Sequential Organ Failure Assessment score, can also be reflected by the proportional increase in HBP. Consequently, HBP could be a helpful and promising biomarker for sepsis and infection.

## 1. Introduction

The heparin-binding protein (HBP) is an enzymatically inactive serine protease with antimicrobial activities [1]. Its uniqueness lies in its behavior pharmacokinetically because it is released from activated neutrophils acutely in response to stress, even before other common biomarkers, including C-reactive protein (CRP) and Procalcitonin (PCT) [2,3,4,5]. Regarding sepsis, according to the sepsis 3 definition, HBP can serve as a diagnostic tool and can also reflect sepsis-related organ damage, as it has been found to correlate with SOFA score [3,4]. Recent reviews highlight that although CRP and PCT remain widely used, several novel biomarkers such as presepsin, monocyte distribution width (MDW), interleukin-10 (IL-10), and HBP have gained attention, while research is expanding into RNA-based biomarkers, oxidative stress-related markers, and extracellular vesicle signatures. These advances reflect a shift toward multi-marker panels and personalized approaches, although variability and lack of standardization remain important challenges [6,7]. In the setting of evidence-based medicine, while there is no ideal biomarker and sepsis is a life-threatening condition with emerging morbidity and mortality due to the increase of multidrug-resistant pathogens and the worldwide decrease in healthcare systems’ support and funding, there is vital essence in comprehensive research of every possible tool that could help clinicians. The scope of this review is to focus on HBP pathophysiology and stepwise to analyze its utility as a sepsis biomarker.

## 2. Methods

A PubMed search was conducted between November 2024 and February 2025 using the following terms: ((heparin binding protein OR HBP) and (sepsis OR pathophysiology OR prognosis OR diagnosis)). All types of studies were potentially eligible for inclusion in this review. We excluded expert opinions, studies referring to the child population, and animal studies. After deduplication, a two-step screening process was conducted independently by three authors. Relevant data were extracted from each included study in a pre-defined table format (first author, publication year, aim of the study, study design, results of the study, conclusions including prognostic and diagnostic evaluation).

## 3. Key Points

### 3.1. Heparin Binding Protein Pathophysiology

- HBP is an enzymatically inactive serine protease, mainly stored in azurophilic granules and rapidly released during infection or stress, often preceding the rise of other inflammatory biomarkers.
- HBP actively contributes to sepsis pathophysiology by recruiting monocytes, increasing endothelial permeability, promoting vascular leakage, and enhancing pathogen clearance.

### 3.2. Biomarkers in Sepsis

- Established biomarkers, including lactate, CRP, PCT, WBC, NLR, and PSP, are valuable diagnostic and prognostic tools for sepsis, but are limited by specificity and response timing.
- HBP emerges as a promising biomarker with potential to enhance early diagnosis and risk stratification in sepsis.

### 3.3. HBP as Biomarker

- HBP demonstrates high diagnostic and prognostic accuracy across multiple bacterial infections—including central nervous system, urinary tract, respiratory, and postoperative infections—often outperforming or complementing traditional biomarkers.
- Beyond bacterial infections, elevated HBP levels are associated with viral infections such as COVID-19, as well as pancreatitis, cardiothoracic surgery, and other inflammatory conditions, reflecting its broader clinical utility.

### 3.4. HBP and Sepsis

- HBP demonstrates superior diagnostic and prognostic accuracy for sepsis, septic shock, and organ dysfunction compared to traditional biomarkers, with elevated levels detectable up to 72 h before clinical deterioration, supporting timely initiation of sepsis bundle therapies.
- Dynamic changes, integration of HBP with clinical scores, and cutoff thresholds of HBP significantly enhance mortality prediction, severe sepsis progression, and differentiate conditions such as COVID-19-related sepsis and autoinflammatory diseases.

## 4. Discussion

### 4.1. Heparin Binding Protein Pathophysiology

HBP or azurocidin or antimicrobial protein 37 is a 29 kDa cationic protein that belongs to the serine protease family but is enzymatically inactive due to a mutation in the catalytic center. Even though the catalytic triad of most serine proteinases is formed from His, Asp, and Ser, in HBP, His 41 and Ser 175 are replaced by Ser and Gly, respectively [1]. HBP consists of 221 or 222 amino-acid residues and can be used as a biomarker for identifying patients with a high risk of developing sepsis, as studies have validated that its levels are rising in the plasma of patients experiencing hypotension or organ dysfunction [8].

As part of the innate immunity, HBP is released in response to infections, especially those with sepsis and septic shock [3,9]. However, various non-infectious but inflammatory stimuli, such as burns, trauma, surgery, and cardiac arrest, may be associated with HBP release [10]. For example, cardiac surgery can cause HBP elevation without infection, especially in challenging circumstances. False-positive elevation is observed in hemolyzed samples [11]. Consequently, this could narrow HBP’s specificity in infected patients with the aforementioned comorbidities. The largest amount of HBP is performed in neutrophils and stored in the secretory and azurophilic granules. Monocytes produce only a small proportion of total HBP [12]. During infection, activated neutrophils rapidly secrete HBP (89% in the first 30 min) in the presence of cytokines and bacterial antigens [12,13,14,15,16]. Moreover, certain pathogens (*Streptococcus pyogenes*, *Streptococcus suis*, *Staphylococcus aureus*, *Escherichia coli*, *Leptospira* spp.) and infectious diseases (erysipelas, necrotizing fasciitis, acute bacterial meningitis, chronic ulcer) are known for causing elevated HBP levels [12]. Interestingly, HBP undergoes exocytosis from secretory vesicles earlier than azurophilic granules [13,17]. Additionally, the secretion of HBP precedes the release of other inflammatory markers, such as PCT, CRP, tumor necrosis factor-α (TNF-α), interleukin-6 (IL-6), and interleukin-8 (IL-8), occurring earlier than the onset of hemodynamic stability and organ dysfunction [2,3,4,5]. However, many studies have pointed out that serial measurements of HBP provide more diagnostic and prognostic information about sepsis than a single measurement in the emergency department or shortly after admission to the hospital [18,19]. The liver primarily mediates the distribution and metabolism of HBP, with a minor contribution from splenic macrophages [20].

**Figure 1:**
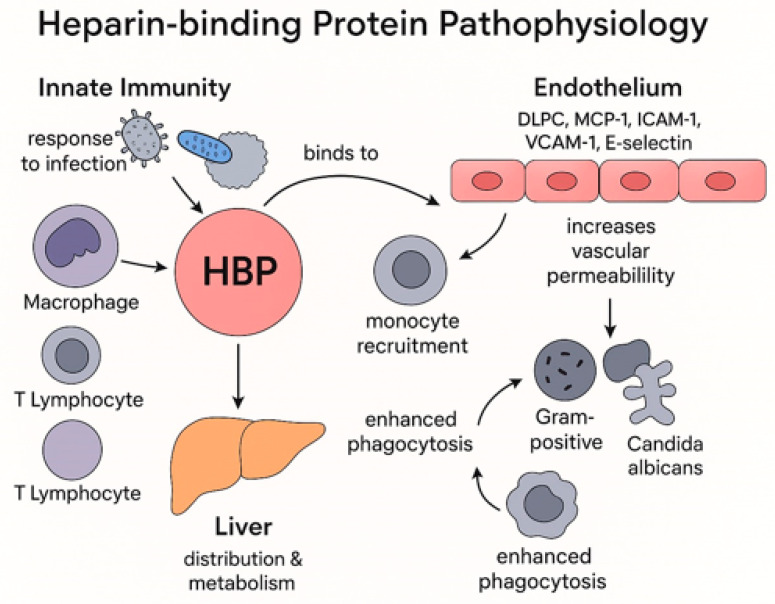
HBP pathophysiology and its role in innate immunity and response to infection. DLPC: 1,2-Dilauroyl-sn-glycero-3-Phosphocholine, MCP-1: Monocyte Chemoattractant Protein-1, ICAM-1: Intercellular Adhesion Molecule 1, Vascular Cell Adhesion Molecule 1.

After its activation, the protein stimulates many cells of the immune system, such as macrophages, T lymphocytes, and neutrophils, which participate in the immune response, and recruits them to target the infection. Initially, HBP functions as a monocyte-specific chemoattractant, as it directly activates the C-C motif chemokine receptor 2 (CCR2), present on the surface of monocytes, which leads to monocyte recruitment, propagates inflammation, and causes tissue damage. Additionally, HBP binds to the endothelial cell-surface proteoglycans and affects the ECs [21]. This connection increases endothelial permeability and upregulates the expression of MCP-1, ICAM-1, VCAM-1, and E-selectin in ECs and thereby enhances monocyte adhesion to the endothelium [22]. The binding consequently activates protein kinase C and rho kinase and promotes calcium influx into the cells, which leads to cytoskeletal rearrangement, creating gaps in the endothelium, causing vascular leakage [23,24]. Furthermore, HBP interacts directly with pathogens, acting as an opsonin factor to enhance the phagocytosis of Gram-positive and Gram-negative bacteria, as well as *Candida albicans* [25]. Figure 1 summarizes HBP pathophysiology.

### 4.2. Biomarkers in Sepsis

In the modern world, sepsis is a leading cause of death, and its various clinical and pathophysiological phenotypes and symptoms render the diagnosis challenging [26]. The early diagnosis of septic shock increases the probability of applying the most appropriate therapy and achieving a positive outcome [27]. This is the reason for the interest in using biomarkers as tools to enable early recognition of infection and to predict its complications [28].

It is important to analyze some frequently used biomarkers in order to better understand the role of HBP as a biomarker. Lactate is an important biomarker because, according to the sepsis definition after the Third International Consensus Definitions for Sepsis and Septic Shock, a serum level of lactate more than 2 mmol/L in a patient with persisting hypotension and in need of vasopressors can be identified with sepsis [29]. Another widely used biomarker is CRP. CRP is produced as a response to an infection or trauma, upregulated by cytokines, especially IL-6, but also IL-1 and TNF [30]. Its normal levels in the blood plasma are usually under 10 mg/L, but CRP’s levels rise rapidly within the first 4–6 h upon inflammatory circumstances, doubling every two hours and peaking between 36 and 50 h, reaching levels up to 400 mg/L [31,32]. This rapid and huge augmentation makes it stand out as a useful biomarker to distinguish inflammation, but also to monitor exacerbations in chronic inflammatory conditions like rheumatoid arthritis [33]. High levels of CRP, as 50 mg/L and more, are related to sepsis and directly associated with organ failure, long duration of ICU stay, and death [34,35]. As far as the connection between CRP and antibiotic treatment is concerned, a systematic review and meta-analysis suggest that treatment is necessary when CRP > 100 mg/L, but not recommended when CRP is <20 mg/L [36].

Furthermore, PCT levels rise within the first 2–4 h of an infection and peak within the first 24 h. It has the advantage of an earlier diagnosis compared to CRP, as well as tracking the recovery progress because its levels normalize earlier than CRP levels [37].

PCT has also been studied for its efficiency in antibiotic treatment guidance. Specifically, clinical trials have demonstrated that utilizing PCT levels to guide antibiotic therapy can reduce prescription rates by up to 72%. So, the use of antibiotics is recommended only when PCL levels are higher than 0.25 μg/L [38].

White Blood Cell count (WBC) is another biomarker to monitor infection, and specifically, leukocytosis (>12,000/mm^3^) or leukopenia (<4000/mm^3^) are a fundamental component of the systemic inflammatory response syndrome (SIRS) criteria [39]. But the WBC count alone has a modest sensitivity and specificity because of its nonspecific elevation in inflammatory conditions or because it may not even rise to abnormal levels and usually is used in combination with other biomarkers [40,41]. Because of this insufficiency, other biomarkers have been developed using the results of the complete blood count, such as the neutrophil-to-lymphocyte ratio (NLR) [42], which represents the balance of neutrophil and lymphocyte levels in the plasma and can point out a systemic infection. Its advantage is that it is really cheap and easy to have access to in clinical practice, and the ratio for significantly healthy people remains quite stable around the world, around 1.65 [43,44]. It can be used to suspect a poor outcome because it is shown that the ratio is higher in patients who suffered and died from sepsis, and can be used in emergency care [45,46].

Pancreatic stone protein (PSP) has recently gained attention as a potential biomarker for sepsis and infection. A study demonstrated that it has a sensitivity of 0.88 and a specificity of 0.78 for sepsis diagnosis. Notably, its levels were found to rise up to 72 h before the clinical presentation of sepsis [47]. More research has demonstrated that measuring PSP within the first 24 h after admission can help predict negative outcomes, including sepsis and the necessity to upgrade antibiotic treatment [48]. Furthermore, it can be used to distinguish high-risk patients with SARS-CoV-2 pneumonia that require intensive care and specific ICU patients who will suffer from infection or even in-hospital death after abdominal surgery or acute necrotizing pancreatitis [49,50].

Thus, due to the increase of these biomarkers in serum, not only in sepsis but also in other pathologies, researchers examine different biomarkers in order to be precise in their clinical practice. One of them is HBP, which is going to be discussed further.

### 4.3. HBP as Biomarker

HBP has been studied as a biomarker of bacterial infections. Such infections are a major cause of hospitalization and mortality globally. A systematic review and meta-analysis by Taha et al. demonstrated that HPB offers a high diagnostic value for various bacterial infections, including central nervous system (CNS), respiratory (RTI), urinary tract infections (UTI), and for differentiating them from non-bacterial infections, contributing to appropriate treatment strategies. Specifically, plasma HBP diagnostic accuracy was excellent for bacterial infections (Sensitivity 0.90, Specificity 0.87), while HBP in cerebrospinal fluid (CSF) performed even better (Sensitivity and Specificity of 0.96 and 0.95, respectively), for the diagnosis of bacterial meningitis. In urinary tract infections (UTI), urine-HBP effectively discriminates bacterial from non-bacterial UTI infections at a cut-off value of 32.868 ng/mL with sensitivity and specificity of 0.87 [51]. HBP may also serve as a predictor of bacterial infection in critically ill patients with severe poly-trauma in the ICU, as its levels were significantly higher in the infected group vs. non-infected (*p* = 0.001) [52].

Regarding CNS infections, a cross-sectional study by Kandil et al. reported that both CSF and serum HBP at cut-off levels of 56.7 ng/mL and 45.3 ng/mL, respectively, exhibited an overall 100% diagnostic accuracy for acute bacterial meningitis (ABM), a life-threatening neurological emergency, with mortality that could approach 100% if untreated [53]. Cerebrospinal fluid bacterial culture is the gold standard for etiological confirmation of acute bacterial meningitis, but fails to achieve 100% diagnostic accuracy in such a life-threatening condition [54,55]. Both CSF and serum HBP levels were significantly higher in ABM than in aseptic meningitis, and the levels remained elevated in the patients who received previous antibiotic therapy. Also, this study denoted serum HPB as an essential and non-invasive biomarker for diagnosis and follow-up in comparison with lumbar puncture, a procedure with complications, that may be contraindicated in patients with CNS diseases, elevated intracranial pressure, and coagulopathies [53]. Linder et al. found that HBP levels in CSF samples were significantly higher (*p* < 0.01) in patients with ABM than in those with other central nervous system infections such as viral meningitis, encephalitis, and neuroborreliosis [56]. Other studies presented similar results for the diagnostic and prognostic value of CSF HBP for patients with intracranial bacterial infections, and particularly for identifying Gram-negative bacterial etiology at a cut-off level of 72.34 ng/mL) [57,58]. According to Kong et al., CSF HBP demonstrated superior diagnostic value to CSF, PCT, or lactate in identifying nosocomial meningitis or ventriculitis, including ventriculostomy-related infections (VRIs), suggesting its potential utility in the early detection of bacterial infections. Nevertheless, HBP demonstrated higher sensitivity and negative predictive value compared to lactate, while its specificity was lower. This suggests that CSF HBP is more useful for identifying infection, rarely missing diagnosis, while lactate indicates active infection, with a lower likelihood of misdiagnosis. On the other hand, CSF PCT presented limited diagnostic value and poor sensitivity [59].

Urinary Tract Infections (UTIs) are one of the most common infections leading to hospitalization, with significant morbidity and antibiotic consumption. Kjölvmark et al. concluded that measuring levels of HBP adds to the diagnostic accuracy for identifying UTIs in adult patients. Urine HBP (UHBP) was found to be significantly superior in the detection of UTIs and in distinguishing cystitis from pyelonephritis, compared to other markers such as IL-6 and WBC, and nitrite in the dipstick test. Also, it was perceived that patients with higher concentrations of bacteria in their urine had significantly elevated HBP levels compared to those with lower bacterial concentrations in culture. However, no correlation between HBP levels and different bacterial species was found [60]. On the other hand, Kjölvmark et al. in another study showed that HBP does not perform well to discriminate UTI from asymptomatic bacteriuria in elderly patients compared to IL-6, despite having a higher negative predictive value [61].

Kong et al. after constructing a logistic model using three inflammatory indicators (HBP, CRP and PCT) for predicting severe acute pancreatitis (SAP), a condition that could lead to progressive systemic inflammatory response syndrome (SIRS) and multiorgan failure, showed that HBP (*p* < 0.001), CRP (*p* = 0.001), and PCT (*p* < 0.001) were risk factors for SAP and the combined model of the biomarkers, HBP-CRP-PCT (AUC = 0.963), could effectively discriminate severe pancreatitis from non-severe pancreatitis improving the patient’s outcome [62]. On the other hand, according to Sjöbeck et al., HBP levels were elevated in patients with AP, although no significant correlation between HBP levels and disease severity or the need for intravenous fluid was found [63].

Regarding community-acquired pneumonia (CAP), Yue Meng et al., in a retrospective analysis, revealed that both blood HBP and NLR were reliable independent indicators of the 30-day prognosis in CAP patients and exhibited superior sensitivity (80.00%) and specificity (76.19%), respectively, for the diagnosis of severe pneumonia. However, no correlation between HBP and the etiology of CAP was found. The potential value of plasma HBP in the severity of CAP and its prognosis is also supported by the favorable relation between plasma HBP and lactate and SOFA score, which have been shown to play a significant role in critically ill patients [64]. Similar findings were observed in other studies regarding the value of HBP in bronchoalveolar lavage fluid (BALF) for the diagnosis of ventilator-associated pneumonia (VAP) [65]. A cohort study by Mellhammar et al. denoted HBP as a predictor of the development of COVID-19-induced organ dysfunction with an AUC of 0.88 when measured within 72 h after admission by the point-of-care (POC) device. Herein, HBP as an inducer of inflammation and vascular leakage seems to play a potential causative role in the onset of organ dysfunction in COVID-19 and therefore could be a potential therapeutic target, although this remains speculative as direct preclinical or clinical evidence is still lacking [66]. Low-molecular-weight heparin (LMWH), which is now routinely administered to hospitalized COVID-19 patients to prevent clot formation, could have an off-target benefit by modulating HBP activity, but this requires further investigation [67]. Saridaki et al. also observed that HBP levels were elevated in severe COVID-19 pneumonia and, when combined with IL-6 at respective cut-off values of 35 and 18 ng/mL, could predict the likelihood of severe respiratory failure and 28-day outcome [68]. According to studies, HBP, IL-1β, and IL-8 were found to be useful markers for diagnosing pulmonary infection in lung-transplant patients and distinguishing from rejection [69], while high levels of HBP are also correlated to acute exacerbation of interstitial lung disease (AE-ILD) [70]. Elevated HBP levels have been found in some severe viral infections, including Influenza A (H1N1) [71].

In the context of cardiothoracic surgery, HBP levels increase significantly following the use of cardiopulmonary bypass (CPB) but normalize rapidly after CPB cessation [8,72,73,74], allowing differentiation between postoperative, surgically induced inflammation and postoperative infection, especially in routine cardiothoracic surgery. HBP concentration was also identified as an independent predictor of increased postoperative complications and 30-day mortality and, corresponding well with previous findings [74,75,76]. High HPB levels were also associated with early death after resuscitation from cardiac arrest, and coronary sinus HBP levels predicted poor outcome in patients with postoperative myocardial injury-related cardiogenic shock (MIRCS) [77]. HBP has also been suggested to be involved in cardiovascular endothelial cell injury and cancers [78,79].

Moreover, HBP seems to be an independent risk factor that can serve as a diagnostic biomarker in patients with obstructive sleep apnea syndrome (OSAS), a condition characterized by an altered systemic inflammatory state, especially neutrophil inflammation [80]. HBP can also be used as a new biomarker for early leptospirosis screening, as leptospires and their secreted products Lsa63 and LipL45 trigger HBP degranulation from human PMNs [81].

Linder et al. also reported that in erysipelas, a skin infection commonly caused by Group A *Streptococcus* (GAS), the levels of HBP were increased in infected skin areas compared to non-infected, possibly due to the interaction of GAS M proteins with neutrophils [82]. HBP has previously been detected in tissue samples taken from patients with GAS necrotizing fasciitis [83].

### 4.4. HBP and Sepsis

According to the Third International Consensus Definitions for Sepsis and Septic Shock (Sepsis-3), sepsis is defined as a life-threatening organ dysfunction triggered by a dysregulated host response to infection, while septic shock represents a subset with profound circulatory and cellular/metabolic abnormalities leading to high mortality [29]. Almost 50 million cases of sepsis and 11 million sepsis-related deaths were recorded globally in 2017, accounting for almost 20% of global deaths [84]. Hence, early recognition of sepsis is essential in order to improve the patient’s outcome, but currently available biomarkers have limitations. Evidence-based guidelines have emphasized scoring systems based on mortality-associated risk factors and serum biological markers as indicators of the context of sepsis [85,86]. However, WBC and CRP are non-specific markers of systemic inflammation, and PCT performs poorly in predicting sepsis-associated organ dysfunction [9,87]. Organ dysfunction in sepsis is typically assessed using the SOFA score, or a simplified version of the quick SOFA (qSOFA) score, in non-ICU settings, but both rely on clinical deterioration rather than early prediction [29,88].

HBP’s diagnostic accuracy in the septic population has been evaluated with multiple meta-analyses reporting broadly consistent findings [3,89,90,91]. In the most recent meta-analysis by Taha et al., who enrolled 28 studies including 5508 patients, primarily using the Sepsis-3 criteria, revealed that HBP demonstrated a Sensitivity of 0.71 and a Specificity of 0.68 in diagnosing sepsis, respectively. Moreover, HPB presented moderate prognostic accuracy for mortality at a cut-off value of 161.415 ng/mL (Sensitivity = Specificity = 0.72), and for severe sepsis outcomes at a cut-off value of 58.907 ng/mL, with a sensitivity and specificity of 71% [89]. HBP diagnostic value for septic patients has been examined by several meta-analyses, which estimate its pooled sensitivity between 0.74 and 0.80 and its pooled specificity between 0.73 and 0.80 [90,91]. Wu et al. described that HBP has superior pooled sensitivity (0.85) to lactate and PCT but similar specificity (0.91), while CRP had the worst diagnostic accuracy [3]. The variability in results between [3,89] meta-analyses is primarily due to differences in study design, patient demographics, and assay methods. Wu et al. demonstrated higher pooled accuracy with low heterogeneity (I^2^ = 12%) by including studies with stricter sepsis definitions (primarily Sepsis-3) and relatively homogeneous populations, predominantly hospital-based adults in ED and ICU settings, with a geographic focus on Asia (notably China) and some Western cohorts. In contrast, Taha et al. reported moderate accuracy with high heterogeneity by using a broader range of studies, pooling both SIRS- and Sepsis-3 defined patients and encompassing more geographically and clinically diverse populations across multiple regions, settings (ED, ICU, mixed wards), and disease severity. Neither meta-analysis specified assay platforms in detail; however, Taha et al. applied the Youden Index to derive pooled optimal cutoffs, which, when applied across heterogeneous studies, contributed to variability in diagnostic performance.

Additionally, several studies have established a significant correlation between elevated HBP levels and the development of sepsis, particularly in cases with circulatory symptoms. Mechanistically, HPB promotes inflammatory responses, vascular leakage, and endothelial permeability, which are central to the pathophysiology of sepsis and septic shock [92]. Clinically, HPB appears to be an early biomarker of severe sepsis-related complications—including septic shock, respiratory distress syndrome, multiple organ failure, arrhythmias, and disseminated intravascular coagulation (DIC)—with elevated levels detectable up to 12 h before circulatory collapse in septic patients [89,93,94]. This early elevation may therefore guide timely interventions such as fluid resuscitation and antibiotic therapy, which are essential components of sepsis bundle management [93,95,96]. Supporting this, Linder et al. revealed that HBP concentrations exceeding 15 ng/mL were a better indicator of septic shock than PCT, CRP, or WBC (AUC = 0.85, sensitivity = 87.1%, specificity = 95.1%) and were also prognostic of organ dysfunction [93]. HPB’s release occurs prior to other inflammatory biomarkers and hence demonstrates superior ability to predict shock as early as 72 h before its onset, and highly correlates with organ failure [3,92,95,97]. Neither CRP nor lactate has demonstrated these features. On the other hand, Linder et al. demonstrated that a combination of CRP, PCT, WBC, lactate, and HBP had superior predictive ability to detect sepsis than any one biomarker used alone. Other multicenter studies suggested optimal cut-off values of 19.8 ng/mL for risk stratification within 72 h of ED admission and 30 ng/mL for predicting progression to severe sepsis with 78% sensitivity [95]. Kahn et al. similarly confirmed that values above 15 ng/mL were prognostic of organ dysfunction [96].

Beyond single-point measurements, dynamic changes in HBP provide further important prognostic insight. Dou et al. reported a greater predictive accuracy of 48-h HBP (AUC: 0.82) change than baseline HBP (0.79), PCT (0.72), lactate (0.71), CRP (0.65), and HBPc-24 (0.62) hours for the prediction of 30-day mortality in critically ill patients with sepsis in the ICU [13]. Similar findings have been observed for predicting 28-day mortality for patients with septic shock in ICUs by measuring dynamic changes in HBP, in the first 72 h of admission [18]. In line with this, Abdou et al. demonstrated in a retrospective ICU study that sustained HBP elevation over the first three days of admission was strongly associated with sepsis severity and mortality, reinforcing its role as a reliable prognostic biomarker in critically ill patients [98]. In another prospective analysis, it was found that both HBP and D-dimer were positively correlated with SOFA score (*p* < 0.05) and considerably higher in the non-survival group. Their combination achieved superior prognostic performance (AUC = 0.830) compared to either marker alone (AUC = 0.824, 0.77, respectively) in predicting sepsis prognosis [4], aligning with findings from Zhang and Lu [99,100]. Furthermore, incorporating serum HBP levels with qSOFA score at the time of ED admission significantly improved the risk stratification and the 30-day mortality discrimination (AUC, 0.80 vs. 0.70; *p* < 0.001) compared with the qSOFA model alone [5].

The clinical utility of HBP has also been demonstrated in viral infections. In COVID-19-related sepsis, HBP levels were closely associated with disease aggravation, respiratory failure, coagulation abnormalities, and pulmonary injury [66,68]. Notably, HBP levels increased approximately 5 days preceding any imaging features of organ dysfunction, emphasizing the key role of HBP in systemic inflammation and the resemblance between severe COVID-19 and bacterial sepsis [101,102].

Moreover, in clinical settings, a significant association has been observed between HBP levels and the development of sepsis-induced acute kidney injury (AKI), with HBP promoting inflammation of renal epithelial cells, suggesting a potential causative role in the pathophysiology of AKI [97].

HBP seems to be also a useful biomarker to differentiate sepsis from various systemic autoinflammatory conditions, including Adult-onset Still’s disease (AOSD), and to determine disease activity in patients with AOSD at the cutoff value of 65.10 ng/mL, with higher levels indicating sepsis, and lower levels indicating AOSD [103].

The diagnostic and prognostic utility of HBP has been demonstrated across diverse types of sepsis, including bacterial and viral sepsis. Table 1 and Table 2 summarize all important studies regarding HBP and sepsis.

## 5. Conclusions

The heparin-binding protein is a promising biomarker for sepsis and infection for both causative differential diagnosis and prediction of complications. Despite several biomarkers being studied in this concept, none of them performs excellently in every dataset and condition. Thus, the importance of disquisition on each of them to better understand its role and position in the process of sepsis and how it is related to clinical outcomes will help clinicians to identify and establish the role and place for each of them and properly use them to answer essential questions in their patients’ clinical course. HPB could serve as an early detector of sepsis and stratify patients causatively and according to their organ dysfunction risk. If current challenges-such as the cost and accessibility of rapid point-of-care tests and variability across laboratory platforms—are overcome, HBP could be more readily integrated into routine clinical practice and improve patient outcomes. Nevertheless, further investigation is needed through larger randomized trials to validate previous findings from observational and small-scale studies and to assess the practical clinical applications of HBP as an effective biomarker. With continuing research and technological advances, HBP has the potential to become a key tool in guiding timely diagnosis and personalized management of sepsis.

## Figures and Tables

**Table 1:** Summary of studies regarding the HBP diagnostic role in sepsis.

Diagnostic Role of HBP in Sepsis				
First Author	Publication Year	Study Type	Major Findings	Sample Size
Taha, A.M. [89]	2024	Systematic review and meta-analysis	HBP presented moderate diagnostic value in sepsis validating its use as a complementary tool alongside established clinical assessments and other well-studied biomarkers. Sensitivity of 0.71 and a specificity of 0.68.	The meta-analysis enrolled 28 studies including 5508 patients.
Wu, Y.L. [3]	2021	Systematic review and meta-analysis	HBP demonstrated high diagnostic accuracy in detecting sepsis among critically ill patients with a pooled sensitivity of 0.85 and specificity of 0.91 outperforming PCT (sensitivity = 0.75, specificity = 0.85) CRP (sensitivity = 0.75, specificity = 0.71) and lactate (sensitivity = 0.64, specificity = 0.80)	The meta-analysis enrolled 26 studies including 3868 patients
Tian, R. [103]	2021	Prospective observational cohort study	Serum HBP differentiates active adult-onset Still’s disease from sepsis at a cutoff of 65.1 ng/mL (sensitivity = 0.759, specificity = 0.552) and from inactive disease at a cutoff of 35.59 ng/mL (sensitivity = 0.650, specificity = 0.889), supporting its potential use as a diagnostic biomarker	In the study were enrolled 30 patients with AOSD, 29 patients with sepsis, and 30 healthy individuals. Of those with AOSD, 20 had active and 10 had inactive disease
Chen, S. [90]	2019	Meta-analysis	HBP demonstrated good diagnostic accuracy for adult sepsis with a pooled sensitivity of 0.74 and specificity of 0.73 and can be used as an auxiliary biomarker in clinical diagnostic protocols.	This meta-analysis included 14 studies with a total of 2023 subjects: 1120 in the sepsis group and 903 in the non-sepsis control group (795 patients with non-sepsis conditions and 108 healthy volunteers).
Chen, M. [91]	2019	Systematic review and meta-analysis	HPB presented strong diagnostic performance in adult sepsis, with both pooled sensitivity and specificity of 0.80	In this meta-analysis 10 studies with 1884 patients were included.

**Table 2:** Summary of studies regarding the HBP prognostic role in sepsis.

Prognostic Role of HBP in Sepsis				
First Author	Publication Year	Study Type	Major Findings	Sample Size
Taha, A.M. [89]	2024	Systematic review and meta-analysis	HBP demonstrated moderate prognostic accuracy for mortality at a cutoff value of 161.415 ng/mL, with a sensitivity and specificity of 0.72 and for severe sepsis outcomes at a cutoff value of 58.907 ng/mL, with a sensitivity and specificity of 0.71.	This meta-analysis included 28 studies with 5508 patients.
Abdou, K.M. [98]	2024	Retrospective observational study	Sustained HBP elevation during the first 3 days strongly correlated with sepsis severity and mortality, confirming its reliability as a prognostic biomarker. The AUC for HBP at day 3 was 0.831 (*p* = 0.000). and at a cut-off value of > 9.5 ng/mL, sensitivity was 0.83 and specificity was 0.77	This study enrolled 55 adult patients who have been proven to have sepsis, and were hospitalized into the intensive care unit.
Tang, J. [4]	2023	Prospective observational cohort study	HBP and D-dimer exhibited significant prognostic value in septic patients, and their combined use—particularly alongside SOFA scores—enhanced the accuracy of 28-day mortality prediction. The AUC of their combination was 0.824, and the sensitivity and specificity were 0.68 and 0.92, respectively.	The study involved 51 ICU patients diagnosed with sepsis, who were monitored over a 28-day period to assess the prognostic value of serial HBP, D-dimer levels, along with the SOFA score.
Xue, H. [18]	2023	Prospective observational cohort study	Dynamic changes in HBP, PCT, and CRP within 72 h of ICU admission significantly predicted 28-day mortality in septic shock patients, with HBP and PCT levels being significantly higher in non-survivor during this period (*p* < 0.001)	In the study, 146 patients with septic shock admitted to ICU, of whom 82 and 64 were survivors and non-survivors, respectively.
Dou, Q.L. [13]	2022	Prospective observational cohort study	48-h HBP change (HBPc-48 h) was an independent prognostic biomarker for 30-day mortality in ICU patients with sepsis (AUC = 0.82), outperforming baseline HBP (0.79) as well as PCT (0.72), lactate (0.71) and CRP (0.65)	The study enrolled 206 patients with sepsis or septic shock, including 21 patients who died within 48 h of admission
Han, X. [5]	2022	Prospective observational cohort study	The incorporation of HBP into the quick SOFA score significantly improved 30-day mortality prediction in septic patients (AUC = 0.80) compared with qSOFA model alone (AUC = 0.70)	The study included 794 adult patients who presented to ED with presumed sepsis, classified into three severity groups: survivors, critically-ill, and non-survivors.
Mellhammar, L. [66]	2021	Prospective observational cohort study	Elevated HBP levels measured by a new point- of- care test within 72 h of admission preceded the onset of organ dysfunction in patients with severe COVID-19 supporting its use as an early prognostic biomarker in COVID-19, with an AUC of 0.88 (95% CI: 0.70–1.00, *p* < 0.01)	The study totally enrolled 35 patients with PCR-confirmed COVID-19. For analysis of organ dysfunction risk, 29 patients had blood samples collected within 72 h, among them, 23 developed organ dysfunction and 6 did not
Saridaki, M. [68]	2021	Prospective observational cohort study	Measurement of HBP and IL-6 at hospital admission enhanced early prediction of severe respiratory failure and 28-day mortality in COVID-19 pneumonia patients. For SRF: sensitivity = 0.59, specificity = 0.96. For 28-day mortality: sensitivity = 0.69, specificity = 0.93	The study included 178 patients with SARS-CoV-2–induced pneumonia, who were evaluated using Sepsis-3 criteria and classified into non-sepsis and sepsis group
Xue, M. [101]	2021	Prospective cohort study	Elevated HBP levels were significantly associated with disease aggravation and multiorgan injury in severe COVID-19, highlighting its prognostic value in severe COVID-19.	The study investigated 18 critically ill COVID-19 patients who suffered from respiratory failure and sepsis, compared with 15 age- and sex- matched COVID-19-negative patients with respiratory failure.
Zhang, Z. [99]	2021	Prospective observational cohort study	HBP was positively correlated with SOFA score and organ dysfunction severity (*r* value was 0.60, *p* < 0.01)	The study enrolled 188 patients admitted to the department of emergency of Hunan Provincial People’s Hospital were enrolled. The patients were divided into non-sepsis group (87 patients) and sepsis group (101 patients) according to Sepsis-3 criteria.
Kahn, F. [96]	2019	Prospective, multicenter observational study	HBP predicted impending organ dysfunction within 72 h. AUC for HBP was 0.73 (95% CI 0.68–0.78) among all patients.	The study included a total of 524 emergency department patients, of whom 236 were eventually adjudicated to have a noninfectious disease. 374 had or developed organ dysfunction within 72 h, 54 patients were admitted to an intensive care unit, and 23 patients died within 72 h.
Fisher, J. [97]	2017	Randomized controlled trial	Elevated plasma HBP is associated with development of sepsis-induced AKI, with an AUC of 0.80, suggesting a potential role in its pathophysiology	The randomized multicenter Vasopressin and Septic Shock Trial (VASST) included 296 septic shock patients.
Linder, A. [95]	2015	Prospective international multicenter cohort study	HBP predicted progression to organ dysfunction within 72 h at a cutoff value of 30 ng/mL with a sensitivity of 78.0% and a specificity of 76.3%, outperforming other biomarkers (PCT, WBC, CRP, lactate.	The study included 759 ED patients with a suspected infection. 674 patients were infected, of whom 328 had signs of organ dysfunction within the 72-h study period, including 29 patients with septic shock.
Linder, A. [93]	2009	Prospective observational cohort study	HBP is an early biomarker of circulatory failure in sepsis, at a cutoff of 15 ng/mL with sensitivity = 0.87 and specificity = 0.95 enabling timely identification of patients at risk	In the study were included 233 febrile adult patients with a suspected infection and were classified into 5 groups. 26 patients were diagnosed with severe sepsis and septic shock, 44 patients had severe sepsis without septic shock, 100 patients had sepsis, 43 patients had an infection without sepsis, and 20 patients had an inflammatory response caused by a noninfectious disease.
